# Novel Bat Coronaviruses, Brazil and Mexico

**DOI:** 10.3201/eid1910.130525

**Published:** 2013-10

**Authors:** Luiz Gustavo Bentim Góes, Sicilene Gonzalez Ruvalcaba, Angélica Almeida Campos, Luzia Helena Queiroz, Cristiano de Carvalho, José Antonio Jerez, Edison Luiz Durigon, Luis Ignacio Iñiguez Dávalos, Samuel R. Dominguez

**Affiliations:** Universidade de São Paulo, São Paulo, Brazil (L.G.B. Góes, A.A. Campos, J.A. Jerez, E.L. Durigon);; Universidad de Guadalajara, Jalisco, Mexico (S.G. Ruvalcaba, L.I. Iñiguez Dávalos);; University Estadual Paulista, Araçatuba, Brazil (L.H. Queiroz, C. de Carvalho);; University of Colorado School of Medicine, Aurora, Colorado, USA (S.R. Dominguez)

**Keywords:** bat, coronavirus, Mexico, Brazil, viruses, zoonoses

**To the Editor:** Bats are now recognized as natural reservoirs for many families of viruses that can cross species barriers and cause emerging diseases of humans and animals. Protecting humans against emerging diseases relies on identifying natural reservoirs for such viruses and surveillance for host-jumping events. The emergence of the Middle East respiratory syndrome coronavirus (MERS-CoV) on the Arabian Peninsula (*1*) further justifies increased surveillance for coronaviruses (CoVs) in bats. MERS-CoV most likely is a zoonotic virus from a bat reservoir and is associated with high case-fatality rates among humans. The existence of a diverse array of alphacoronaviruses in bats in the United States, Canada, and Trinidad has been reported (*2*–*6*). Recently, a possible new alphacoronavirus was detected in an urban roost of bats in southern Brazil (*7*), and a survey of bats in southern Mexico reported 8 novel alphacoronaviruses and 4 novel betacoronaviruses, 1 with 96% similarity to MERS-CoV (*8*). These findings expand the diversity and range of known bat coronaviruses and increase the known reservoir for potential emerging zoonotic CoVs. 

Expanding on our previous work (*2*,*3*), we analyzed samples from 97 bats from Brazil and 75 bats from Mexico (Technical Appendix). During 2007–2010, intestinal samples were collected from bats of 10 species in northwest São Paulo state in southeastern Brazil. These bats had been submitted to the University Estadual Paulista for rabies testing as a result of epidemiologic surveillance or, in some cases, because of possible or known contact with humans. During 2011–2012, as part of an ongoing rabies surveillance project, intestinal samples were collected from bats of 12 species in their usual habitats in Jalisco state in midwestern Mexico. Bats from a variety of species, including insectivorous, nectarivorous, frugivorous, and hematophagous bats, were included in this study for the purpose of obtaining a diverse array of potential exposures. Intestines were collected and stored, and RNA was purified as described (*2*). CoV RNA was detected by using a pancoronavirus PCR selective for the RNA-dependent RNA polymerase gene, and amplicons were sequenced as described (*3*). Virus isolation was not attempted as part of this study.

From 1 of 17 *Molossus rufus* bats and 1 of 8 *Molossus molossus* bats, an identical novel alphacoronavirus was detected (BatCoV-*M.rufus*28/Brazil/2010, GenBank accession no. KC886321). Both specimens were collected in Brazil during 2010 from adult male bats that had been found in urban areas on residential property. The 412-nt sequence of this virus was most closely related to alphacoronaviruses detected in *Eptesicus fuscus* bats in North America (82% nt identity), *Myotis australis* bats in Australia (77% nt identity), *Miniopterus* bats in Kenya (77% nt identity), and *Rhinolophus* bats in Hong Kong (77% nt identity) (Figure). Bats of the genus *Molossus* are insectivorous; their geographic range is restricted to the New World, from northern Mexico to northern Argentina.

**Figure:**
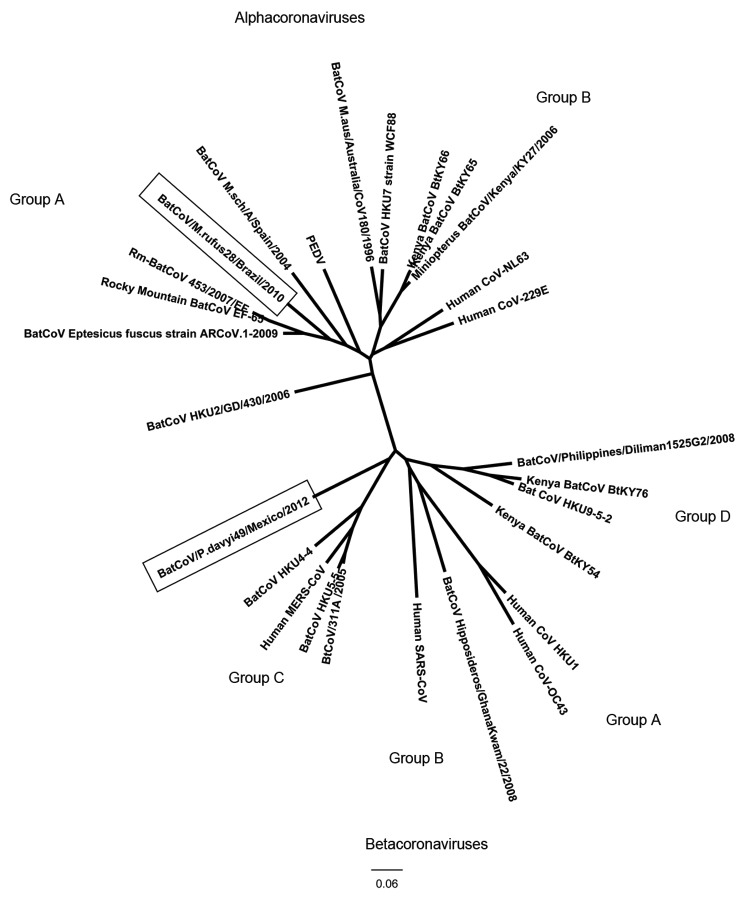
Phylogenetic tree showing relationships based on 412-nt and 439-nt sequences of a conserved region of gene 1b of BatCoV/Molossus rufus28/Brazil/2010 (alphacoronavirus) and BatCoV/Pteronotus davyi49/Mexico/2012 (betacoronavirus) to other known coronaviruses. Sequences were aligned by using ClustalW (www.clustal.org/), phylogenetic analyses were conducted by using the neighbor-joining method and BioEdit (www.mbio.ncsu.edu/BioEdit/BioEdit.html), and trees were constructed by using FigTree version 1.4.0 (http://tree.bio.ed.ac.uk/software/figtree/). Boxes surround the novel alphacoronavirus detected in *Molossus rufus* and *M. molossus* bat specimens from São Paulo state in southeastern Brazil, and the novel betacoronavirus detected in a specimen from a *Pteronotus davyi* bat from Jalisco state in midwestern Mexico. GenBank accession numbers aree AB539081.1, DQ648808.1, GU065420.1, HM211099.1, EF065512.1, EF065512.1, JX869059.2, GU065398.1, EF507794.1, FJ710054.1, JX537914.1, EF544566.1, EU834956.1, EF203064.1, GU065410.1, HQ728484.1, GU065409.1, HQ184049.1, DQ666339.1, HQ336974.1, DQ445911.1, KC210147.1, AY278741, NC_002645.1, and NC_005147.1. PEDV, porcine epidemic diarrhea virus. Scale bar indicates nucleotide substitutions per site.

A novel betacoronavirus (presumably group C) was detected in a specimen from 1 of 4 *Pteronotus davyi* bats (BatCoV-*P.davyi*49/Mexico/2012, GenBank accession no. KC886322). This specimen was collected in 2012 from an adult male bat roosting in a cave in La Huerta, Mexico. The 439-nt sequence of this virus has 71% nt identity to the novel human group C betacoronavirus MERS-CoV and 72% nt identity to various group D betacoronaviruses detected in *Rousettus*, *Pipistrellus*, and *Tylonycteris* bats in the Philippines, China, and Kenya (Figure). Bats of the species *P. davyi* (Davy’s naked-backed bat) are insectivorous and are found from southern Mexico to the northern parts of South America. They prefer to roost in caves and man-made structures, such as mines.

In summary, we found a novel alphacoronavirus in bats from Brazil and a novel betacoronavirus in a bat from Mexico. Both viruses were detected in bats with known or potential contact with humans. Because the bats we sampled were mostly adult males, the prevalence of CoVs that we identified is probably an underestimation of the true incidence of CoVs in these bat populations. For bats of other species, incidence of CoVs among juvenile and female bats is higher (*2*,*9*). Furthermore, we used a non-nested, broadly conserved CoV PCR, which might have limited the sensitivity of CoV RNA detection. The finding of a novel betacoronavirus in insectivorous bats in the New World is noteworthy. Three human CoVs (229E, SARS-CoV, and MERS-CoV) all have animal reservoirs of closely related viruses in Old World insectivorous bats (*10*) from which they most likely emerged, either directly or indirectly, into the human population. Ongoing surveillance for CoVs in wildlife and increased research efforts to better understand the factors associated with CoV host-switching events are warranted.

Technical Appendix Results of reverse transcription PCR analysis of coronaviruses in bats from Brazil and Mexico.
