# Supplementary material for: Novel Bat Coronaviruses, Brazil and Mexico
Source: Emerg Infect Dis. 2013 Oct;19(10):1711–3. doi: 10.3201/eid1910.130525 (PMC3810755; doi:10.3201/eid1910.130525)
Supplement: Technical Appendix — Results of reverse transcription PCR analysis of coronaviruses in bats from Brazil and Mexico. [file 13-0525-Techapp-s1.pdf]

Article DOI: http://dx.doi.org/10.3201/eid1910.130525

Novel Bat Coronaviruses, Brazil and Mexico

Technical Appendix

Technical Appendix Table. Results of reverse transcription PCR analysis of coronaviruses in bats from Brazil and Mexico*

Country, bat species	Diet type	Male, no. (%)	Adult, no. (%)	No. positive/no. sampled (% positive)
Brazil				
Artibeus lituratus	Frugivorous	10 (63)	15 (94)	0/16
Carollia perspicillata	Frugivorous	6 (35)	17 (100)	0/17
Desmodus rotundus	Hematophagous	9 (45)	1 (5)	0/20
Eumops glaucinus	Insectivorous	2 (18)	9 (82)	0/11
Eumops perotis	Insectivorous	0 (0)	1 (100)	0/1
Glossophaga soricina	Nectivorous	4 (100)	4 (100)	0/4
Molossus molossus	**Insectivorous**	**5 (63)**	**8 (100)**	**1/8 (13)**
Molossus rufus	**Insectivorous**	**11 (65)**	**16 (94)**	**1/17 (6)**
Myotis nigricans	Insectivorous	1 (100)	1 (100)	0/1
Noctilio albiventris	Insectivorous	2 (100)	2 (100)	0/2
Mexico				
Artibeus jamaicensis	Frugivorous	1 (50)	Unknown	0/2
Artibeus phaeotis	Frugivorous	1 (50)	Unknown	0/2
Balantiopteryx plicata	Insectivorous	4 (50)	Unknown	0/8
Desmodus rotundus	Hematophagous	21 (81)	Unknown	0/26
Glossophaga soricina	Nectivorous	6 (67)	Unknown	0/9
Lasiurus cinereus	Insectivorous	6 (100)	Unknown	0/6
Mormoops megalophylla	Insectivorous	4 (67)	Unknown	0/6
Pteronotus davyi	**Insectivorous**	**2 (50)**	**Unknown**	**1/4 (25)**
Pteronotus parnellii	Insectivorous	3 (50)	Unknown	0/6
Pteronotus personatus	Insectivorous	4 (100)	Unknown	0/4
Sturnira lilium	Frugivorous	0 (0)	Unknown	0/1
Sturnira ludovici	Frugivorous	0 (0)	Unknown	0/1
Total		102 (59)	75 (76)	3/172 (2)

***Boldface** indicates the novel alphacoronavirus detected in bat specimens from São Paulo state in southeastern Brazil and the novel betacoronavirus detected in a specimen from a bat from Jalisco state in midwestern Mexico.
